# Draft Genome Sequence of *Vibrio* sp. Strain V1B Isolated from the Gut Microflora of the Scallop *Argopecten purpuratus*

**DOI:** 10.1128/genomeA.01130-17

**Published:** 2017-10-19

**Authors:** Wilbert Serrano, Ulrike I. Tarazona, Raul M. Olaechea, Michael W. Friedrich

**Affiliations:** a Escuela de Biología Marina, Facultad de Ciencias Veterinarias y Biológicas, Universidad Científica del Sur, Lima, Perú; b Microbial Ecophysiology Group, Faculty of Biology/Chemistry and MARUM, Center for Marine Environmental Sciences, University of Bremen, Bremen, Germany

## Abstract

A new *Vibrio* strain, V1B, was isolated from the intestinal tract of the scallop *Argopecten purpuratus*. Strain V1B is closely related to the species *Vibrio inhibens* BFLP-10, which has been characterized as showing antagonistic activity against pathogenic *Vibrio* sp. We report here the draft genome of the isolated *Vibrio* sp. strain V1B.

## GENOME ANNOUNCEMENT

The genus *Vibrio* comprises a very successful and versatile group of heterotrophic bacteria that are widespread in marine environments (1). In aquaculture, although first reported as deadly pathogens for scallop larvae (2), some species of the genus *Vibrio* are beneficial (3). Currently, members of this genus have been isolated as symbiotic microflora on the light-emitting organ in squid (4, 5) and characterized as living in association with marine bivalves (6). Here, we report the draft genome sequence of *Vibrio* sp. strain V1B, isolated from the gut microflora of the Peruvian scallop *Argopecten purpuratus*.

Strain V1B was isolated from gut pellets of the scallop *A. purpuratus*, which were collected in Bahia Independencia within the natural protected area in Paracas, Peru (14°14′08.3″ S, 76°11′34.7″ W). Single colonies of strain V1B were obtained from enrichments on alkaline peptone water broth and were further grown on selective thiosulfate citrate bile salts sucrose medium. Genomic DNA was extracted gently according to the Marmur method (7). Genomic insert libraries were prepared using the Nextera XT DNA library preparation kit (Illumina) and sequenced on a MiSeq sequencer (Illumina) at the next-generation sequencing service center GEN LAB SAC (Lima, Peru) using 2 × 250-bp paired-end V3 chemistry. The read library contained 787,448 trimmed paired-end reads with an average coverage of 100×. The quality of the reads was determined with the FastQC tool (8), and sequence trimming was performed using Trimmomatic version 0.32 (9). *De novo* assembly was performed using the SPAdes genomic assembler version 3.10.1 (10) and CLC Genomics Workbench (CLC bio/Qiagen). The draft genome consists of 115 scaffolds with an average length of 50,948 bp. The *N*_50_ value of the assembly was 100,948 bp with a G+C composition of the DNA of 45.1 mol% and genome size of 5,858,972 bp. Gene prediction and annotation were performed using the online Rapid Annotation using Subsystem Technology (RAST) (http://rast.nmpdr.org) server (11). RAST identified 5,223 coding sequences, of which 1,432 were predicted to encode hypothetical proteins and 34 were predicted as noncoding RNAs.

*Vibrio* sp. strain V1B showed a 16S rRNA sequence identity of 99.7% to *V. inhibens* strain BFLP-10, a bacterium isolated from wild long-snouted seahorses (*Hippocampus guttulatus*) and reported as a producer of antagonistic substances against other pathogenic *Vibrio* spp. (3). Strain V1B showed a pool of genes encoding bacteriocins, among which were two marinocines and eight colicins. The presence of the operon *lodAB*, which is responsible for the synthesis of marinocine, within the genome of strain V1B highlights the fact that this organism has the potential to inhibit the growth of similar or closely related strains (12, 13). However, strain V1B apparently lacks known genes involved in the biosynthesis of secondary metabolites, namely, polyketide synthases and nonribosomal peptide synthetases. Moreover, the genome of strain V1B exhibited several gene clusters that may enhance the nutritional capacity of this organism, such as siderophores and carbohydrate-degrading genes, including genes encoding the degradation of chitin.

### Accession number(s).

This whole-genome shotgun project has been deposited at DDBJ/ENA/GenBank under accession number NRQQ00000000. The version described in this paper is the first version, NRQQ01000000.

## References

[1] Thompson FL, Iida T, Swings J 2004 Biodiversity of vibrios. Microbiol Mol Biol Rev 68:403–431. doi:10.1128/MMBR.68.3.403-431.2004. 15353563 PMC515257

[2] Rubi EG, Urbanowsky M, Campbell J, Dunn A, Faini M, Gunsalus R, Lostroh P, Lupp C, McCann J, Millikan D, Schaefer A, Stabb E, Stevens A, Visick K, Whistler C, Greenberg EP 2004 Complete genome sequence of *Vibrio fischeri*: a symbiotic bacterium with pathogens congeners. Proc Natl Acad Sci U S A 102:3004–3009. doi:10.1073/pnas.0409900102. PMC549501 15703294

[3] Balcázar JL, Planas M, Pintado J 2012 *Vibrio inhibens* sp. nov., a novel bacterium with inhibitory activity against *Vibrio* species. J Antibiot (Tokyo) 65:301–305. doi:10.1038/ja.2012.22. 22472571

[4] Prado S, Dubert J, Romalde JL, Toranzo AE, Barja JL 2014 *Vibrio ostreicida* sp. nov., a new pathogen of bivalve larvae. Int J Syst Evol Microbiol 64:1641–1646. doi:10.1099/ijs.0.051417-0. 24510976

[5] Zamborsky DJ, Nishiguchi MK 2011 Phylogeographical patterns among Mediterranean sepiolid squids and their *Vibrio* symbionts: environments drives specificity among sympatric species. Appl Environ Microbiol 77:642–649. doi:10.1128/AEM.02105-10. 21075896 PMC3020525

[6] Romalde JL, Dieguez AL, Lasa A, Balboa S 2014 New *Vibrio* species associated to molluscan microbiota: a review. Front Microbiol 4:413. doi:10.3389/fmicb.2013.00413. 24427157 PMC3877837

[7] Marmur J 1961 A procedure for the isolation of desoxyribonucleic acid from micro-organisms. J Mol Biol 3:208–218. doi:10.1016/S0022-2836(61)80047-8.

[8] Andrews S 2010 FastQC: a quality control tool for high throughput sequence data. http://www.bioinformatics.babraham.ac.uk/projects/fastqc.

[9] Bolger AM, Lohse M, Usadel B 2014 Trimmomatic: a flexible trimmer for Illumina sequence data. Bioinformatics 30:2114–2120. doi:10.1093/bioinformatics/btu170. 24695404 PMC4103590

[10] Bankevich A, Nurk S, Antipov D, Gurevich AA, Dvorkin M, Kulikov AS, Lesin VM, Nikolenko SI, Pham S, Prjibelski AD, Pyshkin AV, Sirotkin AV, Vyahhi N, Tesler G, Alekseyev MA, Pevzner PA 2012 SPAdes: a new algorithm and its application to single-cell sequencing. J Comput Biol 19:455–477. doi:10.1089/cmb.2012.0021. 22506599 PMC3342519

[11] Overbeek R, Olson R, Pusch GD, Olsen GJ, Davis JJ, Disz T, Edwards RA, Gerdes S, Parrello B, Shukla M, Vonstein V, Wattam AR, Xia F, Stevens R 2014 The SEED and the Rapid Annotation of microbial genomes using Subsystems Technology (RAST). Nucleic Acids Res 42:D206–D214. doi:10.1093/nar/gkt1226. 24293654 PMC3965101

[12] Shih-Chun Y, Chih-Hung L, Calvin TS, Jia-You F 2014 Antibacterial activities of bacteriocins: applications in food and pharmaceuticals. Front Microbiol 5:241. doi:10.3389/fmicb.2014.00241. 24904554 PMC4033612

[13] Lucas-Elío P, Gómez D, Solano F, Sanchez-Amat A 2006 The antimicrobial activity of marinocine, synthesized *by Marinomonas mediterranea*, is due to hydrogen peroxide generated by its lysine oxidase activity. J Bacteriol 188:2493–2501. doi:10.1128/JB.188.7.2493-2501.2006. 16547036 PMC1428416

